# Salsolinol: an Unintelligible and Double-Faced Molecule—Lessons Learned from In Vivo and In Vitro Experiments

**DOI:** 10.1007/s12640-017-9818-6

**Published:** 2017-10-23

**Authors:** Magdalena Kurnik-Łucka, Pertti Panula, Andrzej Bugajski, Krzysztof Gil

**Affiliations:** 1 Department of Pathophysiology, Jagiellonian University Medical College, Czysta 18, 30-121 Krakow, Poland; 2 Department of Anatomy and Neuroscience Centre, University of Helsinki, Helsinki, Finland

**Keywords:** Tetrahydroisoquinolines, Salsolinol, Dopamine, Acetaldehyde, DMDHIQ+, Parkinson’s disease

## Abstract

Salsolinol (1-methyl-6,7-dihydroxy-1,2,3,4-tetrahydroisoquinoline) is a tetrahydroisoquinoline derivative whose presence in humans was first detected in the urine of Parkinsonian patients on l-DOPA (l-dihydroxyphenylalanine) medication. Thus far, multiple hypotheses regarding its physiological/pathophysiological roles have been proposed, especially related to Parkinson’s disease or alcohol addiction. The aim of this review was to outline studies related to salsolinol, with special focus on in vivo and in vitro experimental models. To begin with, the chemical structure of salsolinol together with its biochemical implications and the role in neurotransmission are discussed. Numerous experimental studies are summarized in tables and the most relevant ones are stressed. Finally, the ability of salsolinol to cross the blood–brain barrier and its possible double-faced neurobiological potential are reviewed.

## Introduction

Salsolinol (SAL) is a tetrahydroisoquinoline derivative whose presence in humans was first detected in the urine of Parkinsonian patients on l-DOPA (l-dihydroxyphenylalanine) medication (Sandler et al. 1973). Almost side by side, salsolinol was determined in the urine of healthy human volunteers at higher concentrations than that in the urine of intoxicated alcoholics (Collins et al. 1979) and in rat brains treated with ethanol (Collins and Bigdeli 1975). Thus far, multiple hypotheses regarding its physiological/pathophysiological roles have been proposed, especially regarding Parkinson’s disease (PD, 131 records, including 22 reviews in PubMed, April 2017, keywords: “Parkinson’s disease,” “Parkinson disease,” “Parkinson’s,” “salsolinol”) or alcohol addiction (152 records, including 14 reviews in PubMed, April 2017, keywords: “ethanol,” “alcohol,” “salsolinol”).

**Fig. 1:**
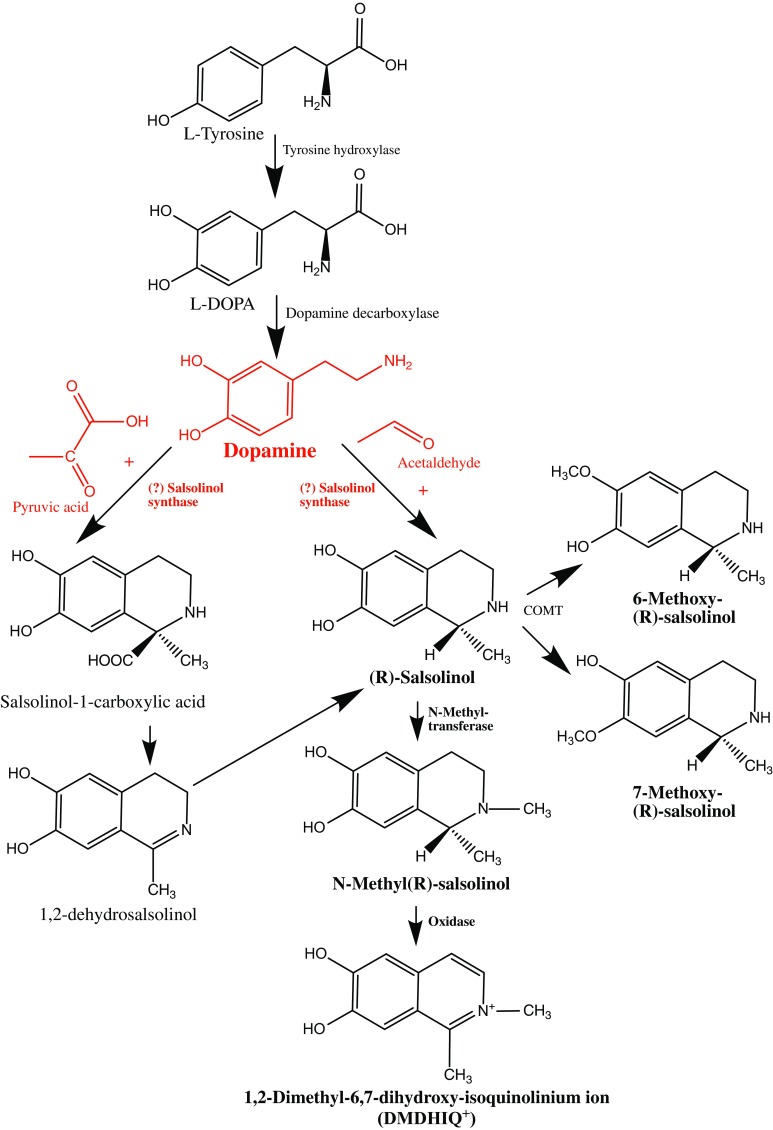
Metabolic pathways of dopamine and (R)-salsolinol in the brain (based on Naoi et al. 2002)

Salsolinol (1-methyl-6,7-dihydroxy-1,2,3,4-tetrahydroisoquinoline) possesses an asymmetric center at C-1; thus, it exists as R and S enantiomers as shown in Fig. 1. Endogenously, non-enzymatic condensation of dopamine (3,4-dihydroxyphenylethylamine, DA) with acetaldehyde yields a racemic mixture of enantiomers (Cohen and Collins 1970), while stereoselective enzymatic synthesis from dopamine via (R)-salsolinol synthase generates the (*R*)-enantiomer (Naoi et al. 1996; Chen et al. 2011). The existence of (R)-salsolinol synthase has been proposed, but its existence may be doubtful because neither has it been isolated and fully characterized nor has its amino acid sequence been determined. Nonetheless, the levels of (*R*)-salsolinol tend to be greater than those of (*S*)-salsolinol in human brain tissue (Deng et al. 1997; Musshoff et al. 1999, 2000, 2003, 2005). Several research groups have quantified both enantiomers in human and rat brain tissue (for a review, see Hipólito et al. 2012), and their presence may indicate a predominance of endogenous synthesis over exogenous accumulation (Musshoff et al. 1999, 2000, 2003, 2005). However, the commonality of salsolinol in many plant- and protein-derived food sources, such as cheese, cocoa powder, bananas, flour, eggs, beer, milk, or fish (Riggin and Kissinger 1976; Riggin et al. 1976; Duncan and Smythe 1982; Duncan et al. 1984; Collins et al. 1990; Deng et al. 1997; examples are given in Table 2), cannot be neglected.

The main aim of this review was to outline the most relevant studies related to salsolinol and focus on in vivo and in vitro experimental models, its possible double-faced (neurotoxic vs. neuroprotective) neurobiological potential and imply its enigmatic role in the periphery.

## Chemical Structure of Salsolinol and Its Biochemical Implications

### Salsolinol and MPTP

The concept of salsolinol contribution to the pathogenesis of idiopathic Parkinson’s disease has emerged from its chemical similarity to MPTP (1-methyl-4-phenyl-1,2,3,6-tetrahydropyridine, Table 1). MPTP is a synthetic compound, still used in industry as a chemical intermediate and is a selective dopaminergic neurotoxin capable of causing parkinsonism in both humans and animals (Langston et al. 1983; Chiueh et al. 1985; for review, see Langston et al. 1987; Singer et al. 1987). However, MPTP should not be considered an etiological factor for Parkinson’s disease while salsolinol being both an endogenous and an environmental compound seems to be a reasonable candidate. The toxicity of MPTP is a result of monoamine oxidase type B (MAO B)-dependent transformation to MPP^+^ ions (Bradbury et al. 1986; Trevor et al. 1988; see Table 1). The MPP^+^ ions can selectively accumulate in dopaminergic neurons due to the activity of the dopamine transporter (DAT) (Javitch et al. 1985), leading to inhibition of the oxidative phosphorylation at complex I of the mitochondrial respiratory chain and reduction of ATP production (Trevor et al. 1987; Singer et al. 1988). The presence of the *N*-methyl group is crucial for MPTP toxicity (Bradbury et al. 1985).

**Table 1:**
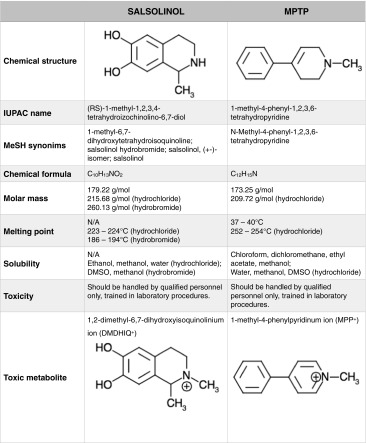
Comparison of the basic physicochemical properties of salsolinol and MPTP (PubChem) ^*N/A* not available, *DMSO* dimethyl sulfoxide^

Indeed, the *N*-methylation of salsolinol into *N*-methyl-salsolinol by *N*-methyltransferase (in fact, by *N*-methyltransferases with different optimum pH values, pH 7.0 and 8.4) was proven in vitro in human brain homogenates (Naoi et al. 1989) and in vivo in the rat brain (Maruyama et al. 1992). *N*-methyl-salsolinol is supposed to be further oxidized into 1,2-dimethyl-6,7-dihydroxyisoquinolinium ions (DMDHIQ^+^) non-enzymatically by autoxidation (Maruyama et al. 1995) or enzymatically by an oxidase sensitive to semicarbazide (Naoi et al. 1995).

### Salsolinol—the Condensation Product of Dopamine with Acetaldehyde

It was hypothesized that, in the brain, the concentration of *N*-methyl-(R)-salsolinol and DMDHIQ^+^ ions depends on the activity of their synthesizing enzymes (Maruyama et al. 1992), but the level of their precursor, (R)-salsolinol, should most likely depend on the dopamine and acetaldehyde (or pyruvate to less extent) concentration in the brain, regardless of how it is synthesized, enzymatically or non-enzymatically. Thus, the following questions, which have not been fully addressed yet, arise: under what circumstances might dopamine (or an excess of dopamine) lead to salsolinol production (and why) and is acetaldehyde produced locally in the brain or from the periphery?

Dopamine (DA), the product of l-DOPA, synthesized indirectly from phenylalanine or directly from tyrosine, is stable in synaptic vesicles. An excess amount of cytosolic DA is easily metabolized via monoamine oxidase (MAO) to produce H_2_O_2_ and dihydroxyphenylacetic acid (DOPAC) or via autooxidation to produce O_2_^−^ and reactive quinones. DA- or l-DOPA-induced neurotoxicity mediated by the generation of free radicals has been reported in damaged neurons both in vitro and in vivo (Cadet and Brannock 1998; Ogawa et al. 1993; Asanuma et al. 2003; Chen et al. 2008; Miyazaki and Asanuma 2008). DA is also broken down by catechol-*O*-methyl transferase and aldehyde dehydrogenase. Together, different breakdown pathways exist, but the main end-product is homovanillic acid (HVA), which has no clearly elucidated biological activity (Eisenhofer et al. 2004). Different cellular regulatory mechanisms and protective biochemical pathways, such as the glutathione pathway, are critical to maintain cytosolic dopamine homeostasis and prevent dopamine-induced neurotoxicity. However, the detrimental effects of dopamine could be exaggerated because of genetic defects or environmental challenges (Chen et al. 2008). For example, parkin-deficient mice showed increased extracellular dopamine levels (measured quantitatively by in vivo microdialysis), likely due to an increase in dopamine release from nigral neurons (Goldberg et al. 2003) while striatal levels of dopamine and its major metabolites (measured by high-performance liquid chromatography in homogenized tissue) were similar between parkin-deficient and wild-type mice. Mutations in the human parkin gene are responsible for autosomal recessive juvenile parkinsonism, a heritable disease that resembles Parkinson’s disease (Goldberg et al. 2003; Perez and Palmiter 2005). Although parkin-deficient mice do not recapitulate signs central to the disease, they might provide important insights into the normal physiological role of parkin in dopamine regulation and nigrostriatal function (Goldberg et al. 2003). Moreover, parkin knockdown in differentiated dopaminergic PC12 cells elevated cellular oxidative stress, endogenous salsolinol and *N*-methyl-salsolinol levels, which were responsible for the higher cell mortality upon exposure to exogenous H_2_O_2_. The results suggest the potential role of salsolinol and its metabolites in parkin knockdown-induced cell vulnerability (Su et al. 2013).

On the other hand, the presence of acetaldehyde is usually associated with alcohol ingestion; however, in fact, rapid metabolism of acetaldehyde by the liver maintains its blood levels extremely low. Even if the blood acetaldehyde levels were significant, either because of genetic variation in alcohol-metabolizing enzymes or certain enzyme inhibitors, acetaldehyde is hardly able to cross the blood–brain barrier (Tabakoff et al. 1976; Westscott et al. 1980; Sippel 1974; Deitrich et al. 2006). However, it has been demonstrated that acetaldehyde can be formed in the brain from ethanol (Aragon et al. 1992; Gill et al. 1992). The intensity of ethanol oxidation is rather low but may be much higher in the specific structures known for their increased catalase activity (Zimatkin and Lindros 1996). The production of acetaldehyde by catalase is limited by the availability of hydrogen peroxide, and acetaldehyde is metabolized to acetate nearly as quickly as it is formed, by aldehyde dehydrogenase (ALDH). ALDH is localized mainly in mitochondria (while catalase in peroxisomes) and thus acetaldehyde can interact with other cellular elements before being further metabolized to acetate (Deitrich et al. 2006). Acetaldehyde can either directly bind to proteins (Jennett et al. 1987; McKinnon et al. 1987; Nakamura et al. 2003), nucleic acids (Wang et al. 2010) and phospholipids (Kenney 1982, 1984; Trudell et al. 1990, 1991) or condense with dopamine and serotonin to form tetrahydroisoquinolines and tetrahydro-beta-carbolines (Deitrich and Erwin 1980; Deitrich et al. 2006). Malondialdehyde or 4-hydroxynonenal produced because of lipid peroxidation could also inhibit the activity of ALDH (Mark et al. 1997; Meyer et al. 2004), which might result in increased levels of acetaldehyde (Deitrich et al. 2006) and might further promote the synthesis of salsolinol in dopaminergic cells (Kim et al. 2016). Increased exogenous salsolinol levels will subsequently elevate the cellular oxidative stress level via releasing reactive oxygen species from mitochondria (Wanpen et al. 2004, see Table 4 for details) and might further potentiate the possible neurotoxic effect of dopamine and l-DOPA.

The direct precursor of dopamine, l-DOPA, can be synthesized either indirectly from the essential amino acid phenylalanine or directly from the non-essential amino acid tyrosine readily available in food, by tyrosine hydroxylase (which action can be inhibited by salsolinol). l-DOPA is converted into dopamine by the aromatic l-amino acid decarboxylase (also known as DOPA decarboxylase), with pyridoxal phosphate as the cofactor. Dopamine is found in many types of food, but it cannot cross the blood–brain barrier; therefore, it must be synthesized locally in the brain. Dopamine can be further used as a precursor in the synthesis of the norepinephrine and epinephrine or can be broken down into inactive metabolites by monoamine oxidase, catechol-*O*-methyl transferase, COMT (both enzymes can be inhibited by salsolinol) and aldehyde dehydrogenase. (R)-Salsolinol synthase possibly catalyzes the reaction of dopamine with acetaldehyde or pyruvic acid to produce (R)-salsolinol or (R)-salsolinol 1-carboxylic acid. Both (R)- and (S)-salsolinol can be also formed non-enzymatically by the Pictet–Spengler reaction of dopamine with acetaldehyde. *N*-Methyltransferase catalyzes the *N*-methylation of (R)-salsolinol, but not that of (S)-salsolinol, into *N*-methyl-(R)-salsolinol. The oxidation of *N*-methyl-(R)-salsolinol can be either non-enzymatic (autooxidation) or enzymatic by an oxidase (sensitive to semicarbazide) and leads to the formation of 1,2-dimethyl-6,7-dihydroxyisoquinolinium ions (DMDHIQ+). Both (R)- and (S)-salsolinol can be metabolized by COMT to form 6-methoxy-(R/S)-salsolinol and 7-methoxy-(R/S)-salsolinol. It remains unknown under what circumstances dopamine might lead to salsolinol production and whether acetaldehyde comes from local production in the brain or from the periphery. It is also uncertain whether exogenous salsolinol (delivered from food) can cross the blood–brain barrier.

The endogenous synthesis of salsolinol, although relatively straightforward, requires appropriate conditions to be maintained. Thus, it seems reasonable to hypothesize that increased salsolinol levels might be rather a consequence of dysregulated enzymatic pathways due to neurodegeneration, genetic mutations, or exogenous inhibitors. Indeed, the peripheral and central catecholamine system acts as the first responder to environmental stimuli. Kocur (2016) reported that the highest levels of endogenous salsolinol in mouse brain striatum homogenates resulted from the intraperitoneal administration of 50 mg/kg ethanol and 1 mg/kg l-DOPA with the combination of MAO (pargyline) and COMT (tolcapone) inhibitors.

### Endogenous Salsolinol and Its Metabolites in Human and Rodent Brain Tissue and Biological Fluids

In the normal human brain, salsolinol (Sjöquist et al. 1981) and *N*-methylated salsolinol (Niwa et al. 1993) have been identified by microdialysis, and the (R)-enantiomers of both salsolinol and *N*-methyl-salsolinol were found to be present (Deng et al. 1995). In fact, both (R)-salsolinol and (S)-salsolinol tend to be present at higher concentrations in areas with increased dopamine synthesis and turnover such as the ventral midbrain and striatum (DeCuypere et al. 2008). The levels of (R)-salsolinol tend to decrease in the caudate, putamen, and substantia nigra according to age (Maruyama et al. 1997). A negative correlation was confirmed between the age and level of *N*-methyl-(R)-salsolinol in the human striatum. The level of a dopamine metabolite, homovanillic acid (HVA), or the ratio of HVA/dopamine, an indicator of dopamine turnover, did not correlate with the levels of catechol isoquinolines in the human brain (Naoi et al. 2004).

For many years, mostly due to analytical methods, it was thought that (R)-salsolinol should be the only enantiomer present in human brain tissue. Some of the analytical difficulties of salsolinol quantification have been recently solved because of the development of more sensitive methods for its analysis (DeCuypere et al. 2008; Rojkovicova et al. 2008; Starkey et al. 2006), hugely improving the detection limits and chiral resolution. However, the lack of agreement among the results reported by different authors (for example DeCuypere et al. 2008 compared with Musshoff et al. 2000, 2003, 2005) is clearly noticeable—for example, 28.6 ng/g ± 18.3 (Musshoff et al. 2000) vs. 204.79 ng/g ± 21.91 (DeCuypere et al. 2008) for (R)-salsolinol and 18.5 ng/g ± 14.1 (Musshoff et al. 2000) vs. 213.19 ng/g ± 25.83 (DeCuypere et al. 2008) for (S)-salsolinol in human substantia nigra. According to Hipólito et al. (2012), these differences could be due to differences in the post-mortem processing of the samples—shorter than 6 h in the DeCuypere study vs. up to 144 h in the Musshoff study. It should also be mentioned that Musshoff et al. (2000) applied gas chromatography/mass spectrometry (GC/MS), while DeCuypere et al. (2008) applied high-performance liquid chromatography with electrochemical detection (HPLC-EC) and liquid chromatography with tandem mass spectroscopy (LC-MS/MS) for (R/S)-salsolinol analysis. Hipólito et al. (2012) elegantly summarized the basal salsolinol levels in different brain areas in healthy humans and alcoholics (see Hipólito et al. 2012).

A low concentration of the racemic form of salsolinol was also detected in normal human cerebrospinal fluid (Moser and Kömpf 1992) and urine (Dostert et al. 1989). Parkinsonian patients treated with l-DOPA and chronic alcoholics showed a significant elevation of the salsolinol concentration in cerebrospinal fluid and urine (Cohen and Collins 1970; Sandler et al. 1973, Collins et al. 1979; Moser and Kömpf 1992), but no difference was shown between de novo Parkinsonian patients and controls (Müller et al. 1999). However, the level of *N*-methyl-(R)-salsolinol in cerebrospinal fluid from untreated patients with PD was significantly higher than that in controls, especially at the early stage of the disease (Maruyama et al. 1996). The activity of a neutral *N*-methyltransferase was found to increase significantly in lymphocytes isolated from PD patients (100.2 ± 81.8 pmol/min/mg of protein) compared that in controls (18.9 ± 15.0 pmol/min/mg of protein) (Naoi et al. 1998). Furthermore, significantly lower levels of (R)-salsolinol, (S)-salsolinol, *N*-methyl-(R)-salsolinol, and *N*-methyl-(S)-salsolinol were found in the caudate nuclei of PD patients compared with those in the normal human brain (DeCuypere et al. 2008).

In rodents, salsolinol concentrations tended to be higher in brain areas rich in dopamine (similarly to humans), and there was a tendency toward an excess of (R)-salsolinol in brain areas with a lower dopamine concentration (Hipólito et al. 2012). In vivo, after the rat striatum was perfused with exogenous (R)-salsolinol, *N*-methyl-(R)-salsolinol was found to occur selectively in the substantia nigra, hypothalamus, and hippocampus, possibly suggesting that the distribution should not solely depend on DA but also on the activity of the synthesizing enzymes. Indeed, the *N*-methyltransferase activity was found to be higher in the rat nigro-striatum than in other brain regions (Maruyama et al. 1992). After the rat striatum was perfused with exogenous *N*-methyl-(R)-salsolinol, *N*-methyl-(R)-salsolinol, and DMDHIQ^+^ ions were accumulated especially in the striatum and, to a lesser extent, in the substantia nigra. Dopamine and noradrenaline levels were reduced in the substantia nigra and in the striatum, whereas serotonin and its metabolites were not affected. In vitro experiments showed that the binding of DMDHIQ^+^ ions to melanin in the substantia nigra was enhanced by Fe(II) whereas Fe(III) enhanced the release of the ions from melanin, and released DMDHIQ^+^ ions could further cause inhibition of the mitochondrial enzymes and depletion of ATP from dopaminergic neurons (Naoi et al. 1996). At the same time, the anti-oxidant and pro-oxidant properties of intracellular dopamine should be noted, especially with regard to their role in the transformation of intracellular iron. Dopamine can form various complexes with both Fe(II) and Fe(III) over a range of pH, further leading to the generation of reactive oxygen species (Sun et al. 2016).

## Role of Salsolinol in Neurotransmission

### Binding Sites for Salsolinol

Selective binding of salsolinol was confirmed not only in brain dopaminergic structures such as the striatum but also in the pituitary gland, cortex, and hypothalamus (Homicskó et al. 2003). Salsolinol produced agonistic effects at muscarinic receptors as well as at α- and β-adrenoceptors (Rodger et al. 1979, see Tables 3 and 4 for details). It produced concentration-dependent inhibition of the vasoconstrictor response to electrical stimulation of the periarterial sympathetic nerves but did not inhibit the vasoconstrictor response to exogenous norepinephrine. The inhibitory effect of salsolinol on neurotransmission was antagonized by yohimbine but not by sulpiride or propranolol. The mono-*O*-methylated metabolites of salsolinol antagonized the inhibition of neurotransmission produced by dopamine. Salsolinol could act as an agonist on prejunctional α-adrenergic receptors and 6-*O*-methyl salsolinol and 7-*O*-methyl salsolinol could act as antagonists on dopaminergic receptors (Nelson and Steinsland 1983).

Salsolinol antagonized the behavioral action of l-DOPA and apomorphine (Ginos and Doroski 1979; Antkiewicz-Michaluk et al. 2000a, b). It suppressed dopaminergic transmission by acting on the agonistic sites of dopaminergic D_1_ and D_3_ receptors different from neuroleptic binding sites (Antkiewicz-Michaluk et al. 2000a, b; Vetulani et al. 2001). Salsolinol did not produce any extrapyramidal symptoms and did not potentiate haloperidol-induced catalepsy in rats (Vetulani et al. 2003).

Several studies have also shown that salsolinol could activate the mesolimbic system. In vitro and in vivo studies (see Tables 3 and 4 for examples) have suggested that salsolinol should exert its action on neuron excitability through a mechanism involving opioid neurotransmission. Salsolinol might act like the endogenous opioid encephalin. In silico analysis predicts a morphine-like interaction between (R)- and (S)-salsolinol with the μ-opioid receptor, and (S)-salsolinol is believed to be a more potent agonist (Matsuzawa et al. 2000; Hipólito et al. 2010, 2011, 2012; Xie et al. 2012; Berríos-Cárcamo et al. 2017). However, there is no direct pharmacological evidence.

[^3^H]-salsolinol also bound specifically to homogenates of the anterior lobe and neuro-intermediate lobe obtained from lactating rats. (R)-salsolinol was present in high concentration in the neuro-intermediate lobe as well as in median eminence extracts of males, and intact and ovariectomized female rats. It was hypothesized that salsolinol could be synthesized in situ and could play a role in the regulation of pituitary prolactin (PRL) secretion, without an effect on the secretion of other pituitary hormones (Tóth et al. 2001, 2002). Salsolinol might regulate the neurotransmission of neuroendocrine dopaminergic neurons by an altered intracellular or intraterminal synthesis and/or distribution of dopamine, thus acting as an endogenous prolactin-releasing factor, especially during lactation (Homicskó et al. 2003; Radnai et al. 2004).

### Catecholamine Transporters

Salsolinol is also regarded as an inhibitor of catecholamine uptake in rat brain synaptosomes because it caused the release of catecholamines stored in the rat brain (Heikkila et al. 1971). In confluent monolayers of human neuroblastoma SH-SY5Y cells, salsolinol at concentrations below 100 μM stimulated catecholamine uptake. Conversely, at concentrations above 100 μM, salsolinol inhibited the uptake of [^3^H]-noradrenaline and [^3^H]-dopamine (Willets et al. 1995, see Table 4 for details).

Takahashi et al. did not find any accumulation of salsolinol in human SH-SY5Y cells mediated by DAT (dopamine transporter) (Takahashi et al. 1994), while Matsubara et al. demonstrated DAT-mediated influx of (R)-salsolinol into rat striatal synaptosomes (Matsubara et al. 1998). However, the affinity of isoquinoline derivatives (especially (R)-salsolinol) for the dopamine transporter compared with MPP+ was proven to be rather low. The intermolecular distance between the *N*-atom and centrinoid of the benzene or catechol ring was suggested as being an important factor in dopamine uptake inhibition (McNaught et al. 1996a). Storch et al. (2002) again addressed the importance of the dopamine transporter molecule for selective dopaminergic toxicity in non-neuronal and neuronal heterologous expression systems of the DAT gene (human embryonic kidney HEK-293 cells and mouse neuroblastoma Neuro-2A cells). The authors concluded that, besides MPP^+^, only the 2[N]-methylated salsolinol derivatives, such as *N*-methyl-salsolinol, showed enhanced cytotoxicity in both DAT-expressing cell lines (Storch et al. 2002).

According to Taubert et al. (2007), in the dopaminergic regions of the substantia nigra, organic cation transporter 2 (OCT2) co-localized with DAT and tyrosine hydroxylase (TH). Cyclo(his-pro) and salsolinol were identified as selective endogenous substrates of the organic cation transporter OCT2. The overlay of energy minimized the conformations (MM2 computation, Chem3D Pro software) of native cyclo(l-his-l-pro) (enol tautomer, endo-conformation), and R-salsolinol uncovered close structural similarities, suggesting a planar ring system, an electrophilic center and a nucleophilic moiety with H-donor properties at a constant distance (of 6.560.3 A °) as key properties of an OCT2-specifc substrate (Taubert et al. 2007).

### Monoamine Oxidase

Nakahara et al. (1994) reported that, following 1 mM (R)-salsolinol perfusion, the dialysate level of 5-HT in the rat striatum markedly increased from non-detectable levels to 4259.2 ± 617.5 nM, while the DA levels increased from 3.4 ± 0.9 to 206.0 ± 56.5 nM. These effects were dose-related to (R)-salsolinol (1 μM to 1 mM) and were confirmed also in three other brain regions (the substantia nigra, the hippocampus and the hypothalamus). The repetitive perfusion with 1 mM (R)-salsolinol into the striatum induced the reproducible response of 5-HT and DA (Nakahara et al. 1994). The analyses of monoamine metabolites in the microdialysate in the rat striatum indicated that salsolinol should inhibit MAO and catechol-*O*-methyltransferase (COMT) activities in situ. Indeed, the levels of 3,4-dihydroxyphenylacetic acid (DOPAC), homovanillic acid (HVA) and, most significantly, 5-hydroxyindolacetic acid (5-HIAA) were reduced (Giovine et al. 1976; Maruyama et al. 1993; Nakahara et al. 1994).

The inhibition of MAO (monoamine: oxygen oxidoreductase EC 1.4.3.4) by salsolinol was first reported by Yamanaka (1971). Salsolinol (racemic mixture) inhibited MAO activity in the rat brainstem and liver homogenates, and the inhibition was competitive to serotonin, a substrate of MAO type A, and non-competitive to benzylamine, a substrate of MAO type B (Meyerson et al. 1976). In vitro, (R)-salsolinol inhibited MAO A more potently than the (S)-enantiomer (Minami et al. 1993). The oxidized DMDHIQ^+^ is the most potent inhibitor of MAO A, followed by *N*-methyl-(R)-salsolinol, (R)-salsolinol, and *N*-methylnorsalsolinol (Naoi et al. 1994). The presence of hydroxyl groups at the sixth and seventh positions and substitution of a hydrogen group at the first position with a methyl or dihydroxybenzyl group are required for the inhibition, whereas the absence of a methyl group or presence of a carboxyl group at the first position, in addition to a methyl group, depletes the inhibitory activity. The structure-activity relationship of isoquinoline derivatives was reported in detail by Bembenek et al. (1990) and Thull et al. (1995). The results were confirmed in vivo (Maruyama et al. 1993).

### Catechol-*O*-Methyltransferase

Salsolinol serves as a substrate for catechol-*O*-methyltransferase (*S*-adenosyl-l-methionine: catechol *O*-methyltransferase, EC 2.1.1.6, COMT) and as a competitive inhibitor (Giovine et al. 1976). Salsolinol was *O*-methylated primarily in vivo at the 7-position, to form salsoline—l-methyl-7-methoxy-6-hydroxy-l,2,3,4-tetrahydroisoquinoline (Collins and Origitano 1983)—whereas catecholamines are *O*-methylated in vivo at the 3-position. The *O*-methylation of catecholamines causes the elimination of their physiological properties, whereas salsoline can still be accumulated and stored in catecholamine nerve terminals of the brain where it might act as false neurotransmitters (Cohen and Mytilineou 1982).

Hötzl and Thomas (1997) reported that *O*-methylation by pig brain-soluble and membrane-bound catechol-*O*-methyltransferase yielded almost equivalent Km and Vmax values for both enantiomers. Their results also indicated that *O*-methylation was stereoselective regarding *O*-methylation patterns: (S)-salsolinol yielded almost equivalent amounts of the two possible 6- and 7-methyl ethers, whereas 7-*O*-methyl derivative was 88% of the product when the (R)-enantiomer was methylated (Hötzl and Thomas 1997, see Table 4 for details).

### Tyrosine Hydroxylase

Exogenous salsolinol also inhibited a rate-limiting enzyme in dopamine synthesis—tyrosine hydroxylase (tyrosine tetrahydropteridine: oxygen oxidoreductase (3-hydroxylating), EC 1.14.16.2, TH)—prepared from rat brain. The inhibition constant, Ki, for salsolinol was 14 μM, and the inhibition was competitive to a cofactor, 6,7-dimethyl-5,6,7,8-tetrahydropterin (Weiner and Collins 1978). The asymmetric center of salsolinol at C-1 plays an important role in changing the enzyme affinity toward l-tyrosine. (R)-Salsolinol deleted the allostery of TH to the biopterin and reduced the activity more markedly than its (S)-enantiomer. The authors suggested that, under physiological conditions, such a conformational change may alter the regulation of DOPA biosynthesis in the brain (Minami et al. 1992).

In vitro, exogenous salsolinol inhibited TH activity in the nanomolar range by binding to both the high and low affinity dopamine-binding sites. Salsolinol produced the same level of inhibition as dopamine when TH was non-phosphorylated. However, it produced 3.7-fold greater inhibition of Ser40-phosphorylated TH than dopamine by competing more strongly with tetrahydrobiopterin. Salsolinol’s potent inhibition of TH phosphorylation might prevent TH from being fully activated to synthesize dopamine (Briggs et al. 2013).

### l-Tryptophan Hydroxylase

Exogenous salsolinol and its derivatives inhibited a rate-limiting enzyme in indoleamine synthesis—tryptophan hydroxylase prepared from the rat brain (l-tryptophan, tetrahydropteridine: oxygen oxidoreductase (3-hydroxylating), EC 1.14,16,2, TPH (l-tryptophan hydroxylase)). The inhibition was non-competitive in terms of either the biopterine cofactor or substrate l-tryptophan. 1,2[N]-Dimethyl-6,7-dihydroxyisoquinolinium (DMDHIQ^+^) ion was an extremely potent inhibitor (Matsubara et al. 1994).

The (R)-and (S)-enantiomers of salsolinol were also found to inhibit the activity of tryptophan hydroxylase prepared from serotonin-producing murine mastocytoma P-815 cells. Inhibition was found to be non-competitive with the substrate l-tryptophan. Tryptophan hydroxylase is composed of two elements with different kinetic properties in terms of the cofactor (6R)-l-erythro-5,6,7,8-tetrahydrobiopterin, and these two elements were inhibited by salsolinol competitively and non-competitively. Thus, salsolinol enantiomers might be naturally occurring inhibitors of indoleamine metabolism (Ota et al. 1992).

### Other Enzymes

Salsolinol exerted a considerable effect on the balance between dopamine and acetylcholine (ACh). (R)-salsolinol and its derivative *N*-methyl-(R)-salsolinol led to concentration-dependent decreases in the activity of acetylcholinesterase (EC 3.1.1.7, AChE). ACh concentrations in the striatum treated with (R)-salsolinol or *N*-methyl-(R)-salsolinol were increased. *N*-methyl-(R)-salsolinol caused a significant decrease in dopamine concentrations, and (R)-salsolinol reduced the concentrations of dopamine metabolites in the striatum (Zhu et al. 2008, see Table 3 for details).

Salsolinol competitively inhibited the activity of debrisoquine 4-monooxygenase in rat liver microsomes, demonstrating that salsolinol has a molecular shape corresponding to the active site of CYP2D1 (Iwahashi et al. 1993, see Table 4 for details).

It was also reported that, when human ceruloplasmin (EC 1.16.3.1, hCP) was incubated with salsolinol, it caused protein aggregation and enzyme inactivation. Reactive oxygen species scavengers and copper chelators inhibited salsolinol-mediated hCP modification and inactivation (Kim et al. 2016, see Table 4 for details). Thus far, decreased hCP ferroxidase activity in cerebrospinal fluid (Boll et al. 1999, 2008) and serum (Tórsdóttir et al. 1999; Bharucha et al. 2008; Martínez-Hernández et al. 2011) from idiopathic PD patients has been reported.

### Clinical Implications

It can be clearly summarized that salsolinol might actively modulate dopaminergic and serotonergic neurotransmission in the brain; thus, it might influence l-DOPA therapy. It was reported by Wąsik et al. (2015) that the acute injection of exogenous salsolinol enhanced the l-DOPA-induced elevation of dopamine release, whereas the chronic administration of salsolinol completely blocked the l-DOPA-induced elevation of dopamine release in the rat striatum. These data demonstrated that the chronic administration of exogenous salsolinol significantly impaired the response of dopaminergic neurons to l-DOPA (Wąsik et al. 2015).

Krygowska-Wajs et al. reported that the concentration of endogenous salsolinol was related to the degree of Parkinson’s disease and cannot be affected by l-DOPA treatment in the cerebrospinal fluid of patients with different degrees of parkinsonism, treated or not with l-DOPA. By contrast, HVA and 3-*O*-methyldopa were significantly elevated in patients receiving l-DOPA but did not correlate with the severity of parkinsonism (Krygowska-Wajs et al. 1997).

## Salsolinol in Experimental Models

The literature on salsolinol’s molecular interactions and its role in neurotransmission is truly multifocal. The presence of two enantiomers and their origin, either endogenous or exogenous, is complex. However, most of the experimental data, both in vivo and in vitro, refer to exogenous salsolinol hydrochloride applied as a racemic mixture. Quintanilla et al. (2014, 2016) not only chirally separated a commercially available (RS)-salsolinol but also purified it from isosalsolinol (isosalsolinol is a by product of non-enzymatical Pictet–Spengler condensation). However, in some studies, salsolinol was synthetized according to different protocols, and its purity was not assessed. Such variations in methodological approaches present a potential barrier to make a comprehensive summary.

### In Vivo Studies

The in vivo models related to salsolinol are summarized in Table 3. Rodent models, especially Wistar and Sprague–Dawley rats, have been the most useful to study the selective occurrence of salsolinol, its metabolism and physiological function, especially in the central nervous system. Salsolinol is regarded as a modulator of dopaminergic neurotransmission, but its exact biological role remains unclear. Thus far, animal modeling has been mainly has been advancing as follows: (1) salsolinol as a modulator of catecholaminergic neurotransmission in the nigrostriatal pathway and possibly as an etiological factor in Parkinson’s disease, (2) salsolinol as a neuromodulator in the mesolimbic pathway related to reinforcing effects of alcohol consumption, and (3) salsolinol as a prolactin-releasing factor in the tuberoinfundibular pathway.

#### Animal Models—the Peripheral Role of Salsolinol?

Mravec et al. (2004) suggested that salsolinol may act at the level of sympathetic ganglia because the intraperitoneal application of salsolinol effectively reduced both plasma epinephrine and norepinephrine levels during stressful situations in rats (Bodnár et al. 2004). Therefore, it was hypothesized that salsolinol might participate in the physiological regulation of the peripheral sympathoadrenal system activity and prevent the over-activation of this system during episodes of acute stress (Mravec 2006). Further research in this matter is also needed.

Unfortunately, most animal studies related to salsolinol have been dealing with its role in the brain, and little attention has been paid to its possible peripheral activity (see Table 3), especially in the enteric nervous system (ENS), which seems quite logical. Catecholamines, such as epinephrine and norepinephrine, are well known to modulate gastrointestinal motility (Li et al. 2004). However, the gut also contains dopamine, and the DA-to-NE ratio in the ENS is higher in the bowel than in other sympathetic targets, and contains a high concentration of the specific DA metabolite 3,4-dihydrioxyphenylacetic acid (Eaker et al. 1988). DA is an enteric neurotransmitter as well. Enteric dopaminergic neurons, which express tyrosine hydroxylase and the dopamine transporter (DAT) but lack dopamine hydroxylase, have been identified in the mouse, guinea pig (Li et al. 2004) and human (Anlauf et al. 2003). The potential of DA (and salsolinol) to influence the gut remains to be fully explored. Until now, it was only shown that exogenous salsolinol induced myenteric neuronal cell (the nitrergic inhibitory motor neurons) death (Kurnik et al. 2015) and altered gastrointestinal motility (Banach et al. 2005, 2006) in Wistar rats. It has never been established if salsolinol could be endogenously formed and further metabolized in the enteric nervous system.

### In Vitro Studies

In vitro models related to salsolinol are summarized in Table 4. The human dopaminergic neuroblastoma SH-SY5Y cell line model has been applied in most of the studies. Salsolinol is attributed to pro-apoptotic activity regardless of the in vitro model. Its toxicity is mostly due to: oxidative damage (formation and release of hydroxyl free radicals) and inactivation of Cu,Zn-superoxide dismutase, with the subsequent disruption of cellular respiration as well as up-regulation of pro-apoptotic and down-regulation of anti-apoptotic proteins. Salsolinol also caused neurofilament (NF-L) aggregation and the loss of glutamate, lysine and proline residues proportional to the concentration and incubation time, as well as induced strand scission and damage in DNA (Table 4). However, all in vitro studies should be always interpreted with caution and verified in vivo.

#### Molecular Mechanisms Related to Salsolinol

Morikawa et al. (1998) reported that salsolinol inhibited most potently mitochondrial complex I activity. Oxidation of *N*-methylated derivatives into *N*-methylisoquinolinium ion augmented the potency to inhibit mitochondrial respiration and complex I (Morikawa et al. 1998). Wanpen et al. (2007) confirmed that mitochondrial complex I activity was significantly decreased, and reactive oxygen species were increased when SH-SY5Y cells were treated with racemic salsolinol. The treatment decreased the levels of the anti-apoptotic protein bcl-2 and increased pro-apoptotic protein bax, while enhancing the release of cytochrome c from mitochondria (Wanpen et al. 2007). Storch et al. (2000) concluded that salsolinol was toxic to human dopaminergic neuroblastoma SH-SY5Y cells by blocking the cellular energy supply via the inhibition of mitochondrial complex II activity (succinate-Q reductase) but not that of complex I. The rapid decrease in the intracellular level of ATP and ATP/ADP ratio of intact cells incubated with salsolinol was dose- and time-dependent (Storch et al. 2000).

Exposure of neuroblastoma SH-SY5Y cells to salsolinol also resulted in a significant decrease in thapsigargin or carbachol-mediated Ca(2+) influx. SH-SY5Y cells treated with salsolinol showed a reduction in transient receptor potential channel 1 (TRPC1) protein levels. Overexpression of the TRPC1 gene and increased TRPC1 protein levels protected SH-SY5Y cells against salsolinol-mediated cytotoxicity. TRPC1 overexpression also inhibited cytochrome c release and decreased the levels of anti-apoptotic bax protein required for apoptosis (Bollimuntha et al. 2006).

*N*-methyl-(R)-salsolinol induced apoptosis in dopamine neurons, as shown in the rat model, and the mechanism of cell death was studied in SH-SY5Y cells (Takahashi et al. 1997; Maruyama et al. 2001, 2002; Akao et al. 1999, Naoi et al. 2002). *N*-methyl-(R)-salsolinol was the most potent to induce DNA damage, whereas *N*-methyl-(S)-salsolinol and salsolinol were less cytotoxic. Apoptosis was initiated by mitochondrial permeability transition as shown by the collapse in the membrane potential, followed mainly by the release of cytochrome c, activation of caspase 3 and final fragmentation of nucleosomal DNA (Maruyama et al. 2001). The enantio-specificity to induce apoptosis was confirmed in isolated mitochondria (Akao et al. 2002). Racemic salsolinol was also cytotoxic to dopaminergic neurons, but a quite different mechanism seems to function in the induction of cell death, mostly due to the production of reactive oxygen species by autoxidation, which resulted in metabolic compromise and necrotic cell death (Storch et al. 2000).

In another in vitro model—the dopaminergic neuronal cell line RCSN-3—salsolinol was found to decrease survival in a concentration-dependent manner. The levels of catalase and glutathione peroxidase mRNA decreased when RCSN-3 cells were treated with salsolinol (Martinez-Alvarado et al. 2001, see Table 4 for details). Neural stem cells (NSCs) cultured from the rat fetal brain challenged with racemic salsolinol elicited a concentration- and time-dependent cell death via the loss of mitochondrial viability. Significant mitochondrial impairment was initiated at 10 μM salsolinol and suggested apoptosis (Shukla et al. 2013, see Table 4 for details). Salsolinol was cytotoxic to rat pheochromocytoma (PC12) cells (Jung and Surh 2001; Kim et al. 2001). Cells exposed to both salsolinol and Cu(II) exhibited higher levels of intracellular reactive oxygen species than those treated with salsolinol alone. These results suggest that copper accelerates the redox cycling of salsolinol, leading to massive production of reactive oxygen species, which can divert the salsolinol-induced cell death to necrosis (Kim et al. 1997, 2001). Salsolinol and *N*-methyl-salsolinol were detected in pheochromocytoma (PC12) cells overexpressing α-synuclein compared with normal PC12 cells and worsened α-synuclein-induced mitochondrial damage (Zhang et al. 2013).

Salolinol and its metabolites impair mitochondrial function in vitro, although the precise mechanisms underlying its toxicity remain poorly understood.

## Salsolinol and the Blood–Brain Barrier

Regarding the determination of the biological potential of salsolinol, an obvious question arises as to whether there is a relationship between salsolinol in the periphery and that in the central nervous system (whether salsolinol can cross the blood–brain barrier)?

Sjöquist and Magnuson (1980) reported that acute intraperitoneal administration of salsolinol to rats resulted in levels of 1–2 nmol/g in the striatum and limbic forebrain after 2 h, whereas the corresponding liver values were about 550 nmol/g, while control animals showed much lower values (Sjöquist and Magnuson 1980; Table 3). However, Origitano et al. (1981) suggested that salsolinol should not be able to cross the blood–brain barrier because single intraperitoneal administration of salsolinol did not result in measurable brain salsolinol or mono-*O*-methyl-salsolinol levels. He also suggested that salsolinol present in the central nervous system of rats during ethanol intoxication should not be of peripheral origin, and salsolinol present in the cerebrospinal fluid of alcoholics during acute detoxification should be formed centrally (Origitano et al. 1981; Table 3). This was further confirmed by Székács using single intravenous administration of ^3^H–salsolinol and who suggested that the target of salsolinol’s action should be at the periphery related to the transport of norepinephrine in the sympathetic nerve terminals (Székács et al. 2007a; Table 3). The data from other recent rodent studies indicate that salsolinol was indeed not able to reach the rat’s brain after a single intraperitoneal administration. Liquid chromatography-tandem mass spectrometry, a sensitive and reliable analytical method (with a detection limit of 2 ng/ml), in combination with in vivo microdialysis was applied for the determination of salsolinol (and other terahydroisoquinolines) in rat brain dialysates (Song et al. 2006a, b; Table 3).

According to Lee et al. (2010), in the long term, salsolinol from different dietary sources (examples are given in Table 2) should be the major contributor to its plasma levels, both in humans and rats. Despite the increases observed in plasma salsolinol or dopamine levels, their levels were not changed in the striatum or nucleus accumbens. The enantiomeric (R/S)-salsolinol and dopamine compositions were determined by a highly specific and reliable method, high-performance liquid chromatography coupled with electrospray ionization-tandem mass spectrometry, with detection limits set at 2 pg for salsolinol isomers and 20 pg for dopamine (Lee et al. 2010).

**Table 2:** Some examples of (R)- and (S)-salsolinol levels in fruits and vegetables obtained in the USA in August 2007, according to DeCuypere (2010). Solid-phase extraction (SPE) was performed on all samples prior to liquid chromatography–mass with tandem mass spectrometry (LC-MS/MS) analysis. Values are expressed in ng/g of wet weight. *SAL* salsolinol

Source	(R)SAL (ng/g wet weight)	(S)SAL (ng/g wet weight)
Mushroom	3572.80 +/− 13.44	3557.40 +/− 17.48
Banana	2717.50 +/− 9.81	2870.87 +/− 10.95
Leaf letuce	2615.23 +/− 42.35	2660.49 +/− 33.55
Celery	1372.85 +/− 15.60	1382.01 +/− 12.03
Grape	951.62 +/− 11.71	980.84 +/− 12.96
Sweet potato	295.23 +/− 5.87	286.80 +/− 8.49
Green bean	195.17 +/− 9.31	215.58 +/− 6.89
Pear	34.09 +/− 1.44	35.18 +/− 9.74
Peach	31.85 +/− 9.51	39.46 +/− 5.54
Cherry	16.73 +/− 3.44	12.03 +/− 4.77

**Fig. 2:**
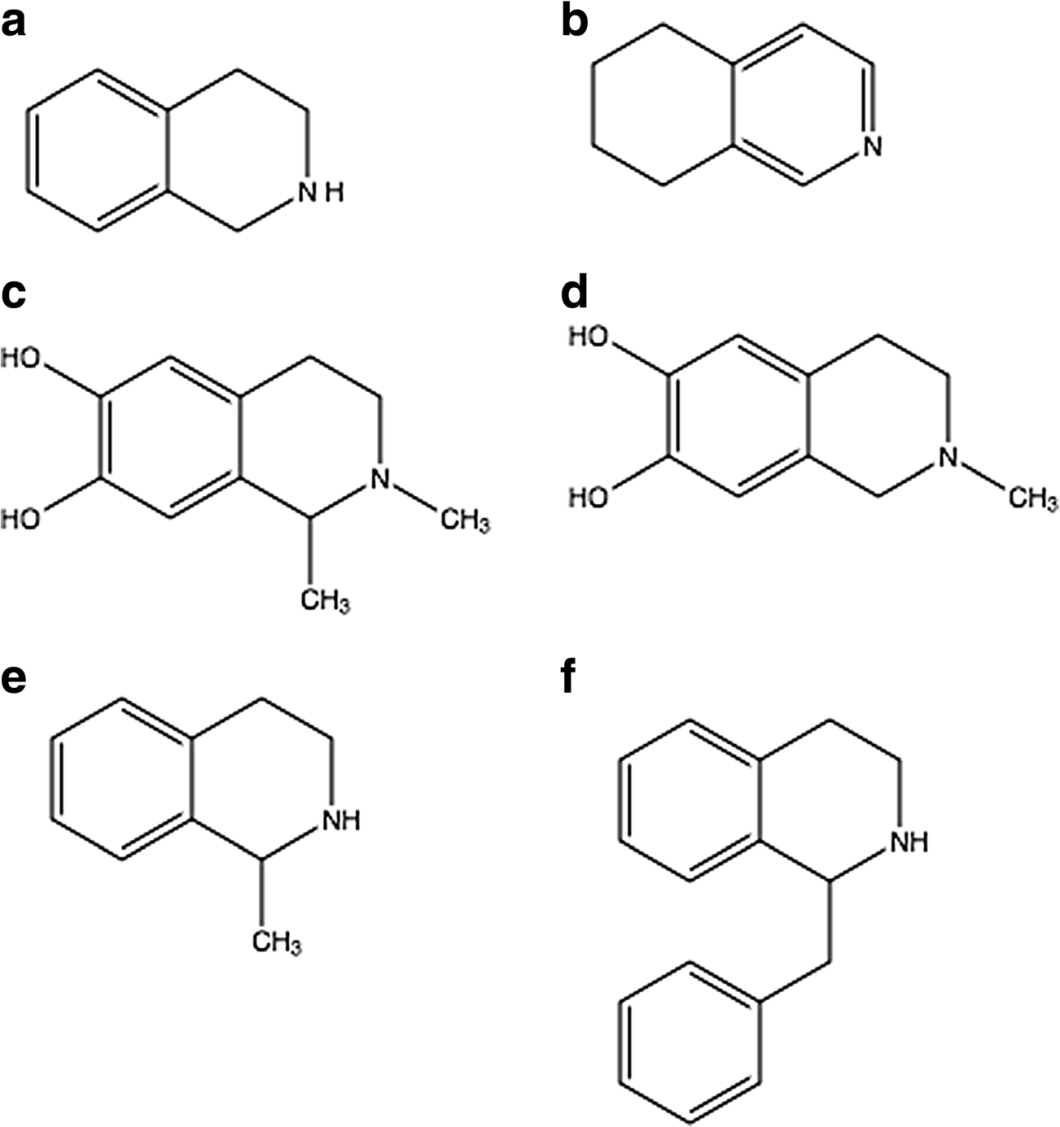
Tetrahydroisoquinolines capable of crossing the blood–brain barrier: **a** 1,2,3,4-tetrahydroisoquinoline, **b** 5,6,7,8-tetrahydroisoquinoline, **c** 1,2-dimethyl-6,7-dihydroxy-1,2,3,4-tetrahydroisoquinoline, **d** 2-methyl-6,7-dihydroxy-1,2,3,4-tetrahydroisoquinoline, **e** 1-methyl-1,2,3,4-tetrahydroisoquinoline, **f** 1-benzyl-1,2,3,4-tetrahydroisoquinoline, shown for structural comparison (ChemDraw Professional 16.0, PerkinElmer Informatics, Inc.)

However, according to Quintanilla et al. (2014), exogenous salsolinol produced conditioned place preference and increased locomotor activity, either injected intracerebrally or intraperitoneally. Following the intraperitoneal administration of salsolinol, this molecule was detected in vivo in the neostriatum and reached an estimated concentration of 100 nM in the dialysate (Quintanilla et al. 2014; Table 3). Several authors have also reported that systemically administered salsolinol can alter laboratory animal behavior (Naoi et al. 1996; Antkiewicz-Michaluk et al. 2000a; Matsuzawa et al. 2000; Vetulani et al. 2001), indirectly suggesting that salsolinol could cross the blood–brain barrier. It was clearly demonstrated that other tetrahydroisoquinoline derivatives (Fig. 2), such as 1,2,3,4-tetrahydroisoquinoline, 5,6,7,8-tetrahydroisoquinoline, *N*-methyl-salsolinol, *N*-methyl-norsalsolinol, 1-methyl-1,2,3,4-tetrahydroisoquinoline, and 1-benzyl-1,2,3,4-tetrahydroisoquinoline, could also cross the blood–brain barrier (Makino et al. 1988; Thumen et al. 2002; Song et al. 2006a, b; Lorenc-Koci et al. 2008). Regarding the physiology of the blood–brain barrier, the most plausible mechanism for *N*-methyl-salsolinol and *N*-methyl-norsalsolinol to cross this barrier should be passive diffusion; thus, salsolinol, with a similar lipophilicity, might be able to diffuse passively through the membranes of vascular cells to enter the brain. However, until now, no study has demonstrated a mechanism through which exogenous salsolinol might cross it. It was successfully demonstrated by Panula et al. (1979) that intraperitoneally injected fluorescent dihydroisoquinoline was trapped in rat brain endothelial cells if administered at a low dose, whereas the injection of a high dose led to leakage to the neuropil in the cerebral cortex and striatum and accumulation in brain cells (Panula et al. 1979). According to Melzig and Zipper (1993), the neurotoxic effect of salsolinol could be caused by damaging endothelial cells associated with a disturbance in the blood–brain barrier (Melzig and Zipper 1993; Table 4).

**Table 3:** Examples of *in vivo* studies related to salsolinol. *DA* dopamine, *i.c.v.* intracerebroventricular, *i.p*. intraperitoneal, *i.v.* intravenous, *N/A* not available, *NAc* nucleus accumbens, *NE* norepinephrine, *PRL* prolactin, *SAL* salsolinol, *TRH* thyrotropin-releasing hormone, *VTA* ventral tegmental area (*a* anterior and *p* posterior part). Salsolinol was applied as a racemic mixture unless otherwise stated

***In vivo*** **rodent models**				
**Concentration and source of salsolinol**	**Route of administration and time course**	**Model and initial body weight**	**Results**	**References**
200 mg/kg b.w. (Sigma-Aldrich, USA) in 0.9% NaCl	i.p. osmotic ALZET minipumps for 2 or 4 weeks	male Wistar rats, 230–265 g	The epididymal fat pad weight over final body mass ratio was lower in SAL-treated rats on high fat diet in comparison with the controls. The area, perimeter, short and long axis of the fad pad adipocytes were significantly decreased in SAL-treated rat.	Aleksandrovych et al. (2016)
			The myenteric neuron count, the mean size of the neuron body, the area of ganglia and the diameter of nerve strands were decreased in both of the SAL-treated groups compared with the controls. Exogenous SAL treatment led to enteric neuronal cell death probably via initiation of apoptosis.	Kurnik et al. (2015)
100 mg/kg b.w.(Sigma-Aldrich, USA) in 0.9% NaCl	i.p. injection once or for 14 consecutive days	male Wistar rats, 220–240 g	SAL under physiological conditions could not be an endogenous factor involved in the neurodegenerative processes, it can rather exert a protective action on nerve cells in the brain.	Możdżeń et al. (2015)
30 pmol (Santa Cruz Biotechnology, USA) in aCSF – purified R and S-SAL	slow injection into the left pVTA	female Wistar-derived naïve UChB rats, 200–250 g	Repeated administration of (R)-SAL caused: (1) conditioned place preference; (2) locomotor sensitization; and (3) marked increase in binge-like ethanol intake; while (S)-SAL did not influence any of these parameters.	Quintanilla et al. (2016)
100 mg/kg b.w.(Sigma-Aldrich, USA) in 0.9% NaCl	i.p. injection once or for 14 consecutive days	male Wistar rats, 220–240 g	Chronic administration of SAL significantly impaired the response of dopaminergic neurons to L-DOPA administration.	Wąsik et al. (2015)
30 pmol/0.2 μL in aCSF for VTA injection or 10 mg/kg in 0.9% NaCl for systemic administration (Santa Cruz Biotechnology or Sigma-Aldrich, USA); free of isosalsolinol	single or repeated injection into the left pVTA or i.p.	female Wistar-derived naïve UChB rats, 200–250 g	SAL produced conditioned place preference and increased locomotor activity, whether intracerebrally or intraperitoneally. Results might indicate that systemically administered SAL is able to cross the blood-brain barrier.	Quintanilla et al. (2014)
0.03, 0.3, 1 or 3 μM (Sigma-Aldrich, USA) in aCSF	single injection into the pVTA	male Wistar rats, 350–400 g	Local application of intermediate concentrations of SAL stimulated DA neurons in the pVTA, whereas higher concentrations may be having secondary effects within the pVTA that inhibit DA neuronal activity.	Deehan et al. (2013)
200 mg/kg b.w. (Sigma-Aldrich, USA) in 0.9% NaCl	i.p. osmotic ALZET minipumps for 2 or 4 weeks	male Wistar rats, 243–263 g	SAL increased serum levels of IL-1β and histamine and the total number of mast cells in the gastrointestinal wall.	Kurnik et al. (2013)
			Diminished body weight gain and lower adipose tissue accumulation in SAL-treated animals were due to delayed gastric emptying together with disturbed gut function resulting in absorptive dysfunction.	Kurnik et al. (2012)
50 mg/kg/day b.w. (Sigma-Aldrich, USA) in 0.9% NaCl	i.p. injections for 3 weeks	male Wistar rats, 180–220 g	SAL proved to be destructive on the mast cells in all segments of gastrointestinal tract	Gil et al. (2011)
30 pmol/200 nL/hemisphere (Sigma-Aldrich, USA) in aCSF	single intra-VTA, bilaterally	male Wistar rats, ~300 g	SAL administered into the pVTA produced psychomotor responses and reinforcing effects, probably, through the activation of μ-opioid receptors.	Hipólito et al. (2011)
0.3, 3, 30, 300, and 3,000 pmol (Sigma-Aldrich, USA) in aCSF	single injection or repeatedly during 12 days into the pVTA	male Wistar rats, 220–300 g	Intra-VTA SAL administration induced an increase of the spontaneous motor activity of the rats with the maximal effect at the dose of 30.0 pmol.	Hipólito et al. (2010)
10 μg of SAL (N/A) in 0.9% NaCl or 3 g of banana (corresponding to 75 μg of SAL) homogenized in 0.9% NaCl	single gavage	male Sprague-Dawley rats; adult male alcohol-preferring (P) and alcohol-nonpreferring (NP) rats, N/A	A single administration of SAL resulted in a significant elevation of rat plasma SAL levels, which declined to near basal levels by 14 hours. The mean plasma levels of (S)- and (R)-SAL at 1 hour after administration were 650 ± 46 and 614 ± 42 pg/ml, respectively. The mean basal (S)- and (R)-SAL levels were 11 ± 4 and 10 ± 1 pg/ml, respectively. A single intake banana also increased the plasma SAL level. Despite the increases observed in plasma SAL or DA levels, their levels were not changed in the striatum or NA. The basal SAL levels were markedly lower in the NA of P than NP rats. The SAL levels in the NA of P rats were not changed after 8 weeks of free-choice alcohol drinking and chronic ethanol drinking did not result in changes of SAL enantiomeric distribution, either.	Lee et al. (2010)
0.1, 5 and 25 μmol (Sigma-Aldrich, USA) in aCSF	single 20-min infusion into shell or core subregions of NAc	male Wistar rats, 300–320 g	Application of 5 and 25 μmol SAL into the core increased the dialysate levels of DA. The administration of the same doses of this drug into the shell significantly reduced the DA levels in this subregion.	Hipólito et al. (2009)
0.03, 0.1, 0.3, 1.0 or 3.0 μM (Sigma, St. Louis, MO) in aCSF with ascorbate	self-infusions into the pVTA or aVTA	male Wistar rats, 250–320 g	SAL produced reinforcing effects in the pVTA of Wistar rats, and these effects were mediated by activation of DA neurons and local 5-HT3 receptors.	Rodd et al. (2008)
10, 20, 40 or 80 nmol (Sigma-Aldrich, USA) in 0.9% NaCl – R-SAL	single injection into striatum	male Sprague-Dawley rats, 250–350 g	(R)-SAL led to a concentration-dependent decrease in the activity of acetylocholinesterase. Acetylocholine concentrations in striatum treated with (R)-salsolinol or N-methyl-(R)-SAL were increased to 131.7% and 239.8% in comparison with control, respectively. (R)-SAL reduced the concentrations of DA metabolites in the striatum.	Zhu et al. (2008)
0.2 to 25 mg/kg b.w. (synthesised at Institute of Pharmaceutical Chemistry, University of Szeged, Hungary) in 0.9% NaCl	single i.p. injection	male and female Sprague–Dawley rats, 250–350 g; male NE transporter knock out (NET KO) mice, 3-5 months old	SAL did not affect the *in vitro* release of DA in the median eminence and did not inhibit the L-DOPA induced increase of DA level in the median eminence. Increasing doses of SAL caused a dose dependent decrease of tissue DA concentration and increase of NE to DA ratio in the salivary gland, atrium and spleen.	Székács et al. (2007a)
25 mg/kg b.w. (synthesised at Institute of Pharmaceutical Chemistry, University of Szeged, Hungary) in 0.9% NaCl		male Sprague–Dawley rats after medullectomy, adrenalectomy and hypophysectomy, 200–300 g	The presence of the adrenal gland was not required for the changes of PRL secretion, nor for the reduction of peripheral sympathetic activity induced by SAL. The effect of SAL on peripheral sympathetic terminals was not affected by hypophysectomy, consequently the role of pituitary hormones in the effect of SAL on the peripheral catecholamine metabolism might be excluded.	Székács et al. (2007b)
1.8 mg/kg b.w. (Sigma–Aldrich, USA)	single i.p. injection	Male Sprague–Dawley rats, 280–320 g	1,2,3,4-tetrahydroisoquinoline (TIQ), 5,6,7,8-tetrahydroisoquinoline (5-TIQ), 1-benzyl-1,2,3,4-tetrahydroisoquinoline (1-BnTIQ), and SAL were studied. TIQ and 5-TIQ passed through the blood–brain barrier more easily than 1-BnTIQ, while SAL was unable to cross the barrier.	Song et al. (2006b)
50 mg/kg/day b.w. (Sigma-Aldrich, USA) in 0.9% NaCl	i.p. injections for 3 weeks	male Wistar rats, ~200 g	SAL had a direct effect on both interstitial cells of Cajal and neuronal pathways of gastro-duodenal reflexes.	Banach et al. (2006)
			Fasting intestinal myoelectrical activity (IMA) recordings did not reveal differences in frequency of migrating myoelectrical complexes and dominant frequency (DF) of slow waves between SAL and saline group. However in response to gastrointestinal stimulation in the SAL group DF of IMA remained unchanged whereas in the controls increased.	Banach et al. (2005)
10 mg/kg b.w. (synthesised at Institute of Pharmaceutical Chemistry, University of Szeged, Hungary) in 0.9% NaCl	single i.v. injection	primiparous lactating Sprague-Dawley-derived rats, N/A	The observed changes in the level of cAMP following the acute treatment of SAL in the median eminence (ME) and the anterior lobe (AL) seems to be related to interacting neuroendocrine signals delivered from the ME to the AL through the long portal vessels to release PRL.	Radnai et al. (2005)
40 mg/kg b.w. (synthesised at Institute of Pharmaceutical Chemistry, University of Szeged, Hungary) in 0.9% NaCl		male Sprague-Dawley rats, 350 g or primiparous lactating female rats, N/A	SAL had an important role in the regulation of PRL release induced by physiologic and environmental stimuli; therefore, it could be considered as a candidate for being the PRL releasing factor in the hypothalamo-hypophysial system.	Radnai et al. (2004)
	i.p. injections	male Sprague-Dawley rats, 350 g	SAL could candidate as an endogenous PRL-releasing factor and a potent inhibitor of stress-induced plasma release of epinephrine and NE.	Bodnár et al. (2004)
1.25 nM (Sigma-Aldrich, USA) in 0.1 M of Tris-HCl	single injection into the right substantia nigra	male Sprague-Dawley rats, 180–220 g	DT-diaphorase played a protective role in the nigrostriatal dopaminergic systems.	Díaz-Véliz et al. (2004)
0.3, 1, 3, 12.5 μM (Sigma, St. Louis, MO) in aCSF ⁄ ascorbate	self-infusions into the shell of NAc	female alcohol-preferring (P) rats from the 49th and 50th generations, 250 to 320 g	SAL was reinforcing into the shell of NA of P rats at concentrations that were pharmacologically possible, and these reinforcing actions were mediated in part by D2/D3-like receptors.	Rodd et al. (2003)
5 mg/kg b.w. in 0.9% NaCl (Sigma, St. Louis, MO)	single i.p. injection	naive male C57BL/6 strain mice 8–9 weeks old; randombred CD-1 mice; male Wistar rats, 220–250 g	SAL antagonized the agonistic conformation of DA receptor and that endogenous 1,2,3,4-tetrahydroisoquinolines may play a role of natural feedback regulators of the activity of dopaminergic system.	Vetulani et al. (2001)
		male Wistar rats, 220–240 g	Acute effects of SAL produced small biochemical effects, did not potentiate the action of DA receptor antagonists, counteracted the action of DA receptor agonists and bound to agonistic sites of DA receptors.	Antkiewicz-Michaluk et al. (2000a)
100 mg/kg b.w. (Sigma-Aldrich, USA) in 0.9% NaCl	single i.p. injection or for 18 days	male Wistar rat, 190–220 g	A single dose of SAL did not affect the DA metabolism in the substantia nigra and NAc, but remarkably increased the homovanillic acid concentration in the striatum (by 55%). The effects of chronic treatment were limited to extrapyramidal structures, and resulted in a remarkable depletion of DA (by 62% in the substantia nigra and by 33% in the striatum), concomitant with the decline of DA metabolites.	Antkiewicz-Michaluk et al. (2000b)
1, 3, 10, 30 mg/kg b.w. (Sigma-Aldrich, USA) in 0.9% NaCl	single i.p. injection	male Sprague-Dawley rats, 170–220 g	SAL might have some rewarding effect, potentiated by psychological stress. The rewarding effect of SAL especially under psychological stress might involve the endogenous central opioid system.	Matsuzawa et al. (2000)
1 mmol (synthetized according to Teitel et al, 1972) in Ringer solution – R and S-SAL	40-min infusion into the striatum	male Wistar rats, N/A	The concentration of serotonin in the rat striatum increased from undetectable level to 2.53 +/- 0.12 and 3.69 +/- 0.01 μmol after perfusion of (R)- and (S)-SAL, respectively. SAL increased extracellular dopamine levels but to a much lesser degree than serotonin.	Maruyama et al. (1993)
20 mg/kg b.w. (N/A)	single i.p. injection	male Wistar rats, N/A	SAL should not be able to cross the blood brain barrier since SAL administered intraperitoneally did not result in measurable brain SAL or mono-O-methyl-salsolinol levels.	Origitano et al. (1981)
0.4 mmol/kg b.w. (N/A)	single i.p. injection	rats, N/A	SAL administration resulted in levels of 1-2 nmol/g in striatum and limbic forebrain after 2 h, whereas the corresponding liver values were about 550 nmol/g. Control animals showed SAL values in liver of about 2 nmol/g and in striatum and limbic forebrain 1 nmol/g tissue.	Sjöquist and Magnuson (1980)
250 μg (synthesized by the method of the Pictet- Spengler condensation of dopamine with an aldehyde) in 0.9% NaCl	single i.c.v. injection	male Wistar rat, 180–250 g	SAL induced rise in striatal dopamine was prevented by alpha-methyl-p-tyrosine pretreatment while SAL induced fall in diencephalic noradrenaline was not affected. SAL was found to cause hypothermia.	Awazi and Guldberg (1979)
0.2-3.0 mg/kg (synthetized according to Craig et al, 1952)	single i.v. injection	adult male and female vagotomised cats, N/A	SAL produced agonist effects at cholinoceptors and alpha- and beta-adrenoceptors. In anesthetized cats, SAL (0.2-3.0 mg/kg) produced dose-related falls in mean blood pressure and a fall in heart rate. These effects were antagonized by atropine (1 mg/kg). In atropinized animals, both SAL caused dose-related elevations in mean blood pressure that were blocked by phentolamine (2 mg/kg). SAL produced a reduction in the tension and degree of fusion of the incomplete tetanic contractions of the soleus muscle, an effect antagonized by propranolol (0.4 mg/kg).	Rodger et al. (1979)
10, 20, 40 or 240 μg (Sigma-Aldrich, USA) in Krebs-Ringer with 0.01% ascorbic acid	single intracisternal injection	male and female mice after 18 generations of genetic selection for alcohol sensitivity, N/A	Low doses of SAL produced significantly lower activity levels in the alcohol-sensitive long-sleep (LS) line than in the alcohol-insensitive short-sleep (SS) line. A hypnotic dose of SAL induced significantly longer sleeptimes in the LS line than in the SS line.	Church et al. (1976)
0.038 or 0.38 mM (Sigma-Aldrich, USA) in aCSF	hippocampal perfusion	adult male and female Sprague-Dawley rats, 300–550 g	SAL enhanced the efflux of ^45^Ca^2+^ in a concentration-dependent manner during the interval of its perfusion within the hippocampal plane.	Myers et al. (1988)
**Other** ***in vivo*** **models**				
**Concentration and source of salsolinol**	**Route of administration and time course**	**Model and initial body weight**	**Results**	**Authors**
1 mg/ml (15 μg/60 μl each infusion) in Ringer–Locke (synthesised at Institute of Pharmaceutical Chemistry, University of Szeged, Hungary)	a series of five 30-min infusions at 30-min intervals to the third ventricle	mature Polish Longwool sheep (3–4 years old), N/A	SAL stimulated oxytocin secretion during lactation in sheep.	Górski et al. (2016)
5 mg/kg b.w. (synthesised at Institute of Pharmaceutical Chemistry, University of Szeged, Hungary) in 0.9% NaCl	single i.v. injection	male Shiba goats, ~25.3 kg	Hypothalamic DA blunted the SAL-induced release of PRL in male goats, regardless of the photoperiod, which suggested that both SAL and DA were involved in regulating the secretion of PRL in goats.	Jin et al. (2014)
		male Shiba goats, ~ 20 kg	DA inhibited the SAL-induced release of PRL in male goats, which suggested that SAL and DA are involved in regulating the secretion of PRL.	Hashizume et al. (2012)
		female Shiba goats, ~27.7 kg	A long photoperiod highly enhanced the PRL-releasing response to SAL in either medium or low ambient temperature in goats.	Yaegashi et al. (2012)
5 μg in total/animal (synthesised at Institute of Pharmaceutical Chemistry, University of Szeged, Hungary) in Ringer-Locke	a series of five 30-min i.c.v. infusions at 30-min intervals	mature Longwool sheep, N/A	SAL might play a role as a neuromodulator for the hypothalamic NE and DA systems and as a signal transmitter for the pituitary PRL release.	Misztal et al. (2011)
50 ng oraz 50 μg in total/animal (Sigma-Aldrich, USA) in Ringer-Locke	a series of five 10-min i.c.v. infusions at 20-min intervals	mature ewes during the second month of pregnancy, N/A	SAL infused at the higher dose significantly increased plasma PRL concentration in lactating ewes. SAL in the process of stimulation of PRL release during lactation and that hypothalamic PRL might play an important role in the central mechanisms of adaptation to lactation.	Górski et al. (2010a)
50 ng oraz 50 μg in total/anaimal (Sigma-Aldrich, USA) in Ringer-Locke		nursing Polish Longwool sheep, 50–55 kg	SAL might affect the regulatory process of growth hormone secretion in lactating sheep but its role might not to be major.	Górski et al. (2010b)
5 mg/kg b.w. (for i.v.) or 10 mg/calf (for IIIv) synthesised at Institute of Pharmaceutical Chemistry, University of Szeged, Hungary) in 0.9% NaCl	single i.v. or i.c.v. injection	Japanese male and female calves, ~144 kg and Japanese Black cows, ~418 kg and castrated Holstein calves, ~204 kg	SAL was involved in the regulatory process for the secretion of PRL, not only in male and female calves, but also in cows. The potency of the PRL-releasing response to SAL differed with the physiological status of cattle.	Hashizume et al. (2010)
5 mg/kg b.w. (synthesised at Institute of Pharmaceutical Chemistry, University of Szeged, Hungary) in 0.9% NaCl	three consecutive i.v. injections at 2 h intervals	Shiba goats, ~27 kg	The mechanism(s) by which SAL released PRL were different from the mechanism of action of TRH. The secretion of PRL was under the inhibitory control of DA and SAL did not antagonize the DA receptor’s action.	Hashizume et al. (2009)
	single i.v. injection	female Shiba goats, ~26 kg	SAL was able to stimulate the release of PRL in ruminants. The additive effect of SAL and TRH on the release of PRL detected *in vivo* might not be mediated at the level of the AP but that DA was able to overcome their releasing activity both *in vivo* and *in vitro.*	Hashizume et al. (2008a)
5 or 10 mg/kg b.w. – for i.v. or 1 or 5 mg/calf – for i.c.v. (Sigma-Aldrich, USA) in 0.9% NaCl	single i.v. or i.c.v. injection	female Shiba goats, ~15 kg; castrated Holstein calves, ~172 kg	SAL was present in extract of the PP gland of ruminants and had PRL-releasing activity both *in vivo* and *in vitro*.	Hashizume et al. (2008b)

**Table 4:** Examples of *in vitro* studies related to salsolinol. *DA* dopamine, *DAT* dopamine transporter, *EC50* the half maximal effective concentration, *IC50* the half maximal inhibitory concentration, *N/A* not available, *NE* norepinephrine, *PRL* prolactin, *SAL* salsolinol, *TH* tyrosine hydroxylase. Salsolinol was applied as a racemic mixture unless otherwise stated

**Cell-based** ***in vitro*** **models**				
**Salsolinol source**	**Salsolinol concentration(hydrochloride unless otherwise stated)**	**Model**	**Main outcomes**	**References**
Santa Cruz Biotechnology, Dallas, TX, USA	0.01 μM – 1 mM (racemic and purified R- and S-SAL)	commercially cell-based assays, composed by recombinant CHO-K1 cells that overexpress only the human μ-opioid receptor	SAL activated the μ-opioid receptor by the classical G protein-adenylate cyclase pathway with EC50 of 2 × 10−5 M. The agonist action of SAL was fully blocked by the μ-opioid antagonist naltrexone. The EC50 for the purified stereoisomers (R)-SAL and (S)-SAL were 6 × 10−4 M and 9 × 10−6 M, respectively. Molecular docking simulations predicted a morphine-like interaction of (R)-SAL and (S)-SAL stereoisomers with the μ-opioid receptor and favoured the interaction for the (S)-SAL stereoisomer.	Berríos-Cárcamo et al. (2017)
N/A	0–1000 μM	human neuroblastoma (SH-SY5Y), human primary glioblastoma **(**U87) and human monocytic (THP-1) cells	SAL was toxic to SH-SY5Y cells in a dose-dependent manner with 47.50% cell death at 500 μM. Similarly, 500 μM SAL induced 13.50 and 50.50% death in U87 and THP-1 cells, respectively.	Wang et al. (2015)
Synthesized (Beijing Institute of Technology, Beijing, China)	275 – 2200 μM	human neuroblastoma (SH-SY5Y) cells	The lethal dose (LD_50_) values for SAL = 1500 μM.	Arshad et al. (2014)
Synthesized (Semmelweis University, Budapest, Hungary)	0–100 μM	bacterial Escherichia coli (BL21 DE-3) cells	SAL completely inhibited DA binding, to both the high and low affinity DA binding sites. The concentration at which half the DA bound was 58 ± 4.4 nM of SAL. It produced 3.7-fold greater inhibition of Ser40-phosphorylated TH compared to DA by competing more strongly with tetrahydrobiopterin.	Briggs et al. (2013)
Sigma Aldrich, St. Louis, MO, USA	100-800 μM	human neuroblastoma (SH-SY5Y) cells	SAL caused a dose-dependent toxicity mediated by apoptosis (increase in caspase-3 levels).	Brown et al. (2013)
	25, 50, 100, 200, 400 and 800 μM		Maximum toxicity (about 50%) was achieved with 400 μM of SAL.	Qualls et al. (2014)
	1–100 μM, especially 10 μM	neural stem cells (NSCs)	Morphological impairment, cleaved caspase-3 and decreased Bcl-2:Bax suggested apoptosis. SAL toxicity coincided with reduced pAkt level and its downstream effectors: pCREB, pGSK-3b, Bcl-2, suggesting repressed PI3K/Akt signaling pathway, confirmed on adding the PI3K inhibitor (LY294002), which abolished the protection	Shukla et al. (2013)
N/A	0 – 400 μM	rat pheochromocytoma (PC12) and parkin knockdown (PC 20) cells	The elevated *parkin* knock down elevated cellular oxidative stress and SAL levels.	Su et al. (2013)
Sigma Aldrich, St. Louis, MO, USA	0–500 μM	human neuroblastoma (SH-SY5Y) cells	SAL neurotoxicity towards SH-SY5Y cells was potentiated during treatment with concentrations of glutathione below 250 μM, whereas glutathione concentrations above 250 μM resulted in protection against SAL-induced neuronal cell death.	Wszelaki and Melzig (2012)
	10–500 μM	human neuroblastoma (SH-SY5Y, SK-NSH) cells	The cell viability decreased in a concentration-dependent manner. 500 μM of SAL caused 49.08 ± 1.8% and 22.5 ± 4.5% cell death in undifferentiated and differentiated SH-SY5Y cells, respectively.	Wszelaki and Melzig (2011)
	250 μM	human neuroblastoma (SH-SY5Y) cells	The anti-apoptotic action of N-methyl-D-aspartate (NMDA) on SAL (250 μM)-evoked cell death in human SH-SY5Y cells was observed, without the influence on caspase-3 activity.	Jantas and Lason (2009)
Synthesized (Szent-Györgyi Albert University, Szeged, Hungary)	0,001 - 10 μM (hydrobromide)	bovine anterior pituitary cells	SAL significantly stimulated the release of PRL from cultured bovine anterior pituitary cells at doses of 1 - 10 μM, compared to control cells.	Hashizume et al. (2008a)
			SAL (1 μM), thyrotropin-releasing hormone (TRH, 0,01 μM) ), and SAL plus TRH significantly increased the release of PRL, but the additive effect of SAL and TRH detected *in vivo* was not observed *in vitro*. DA (1 μM) inhibited the TRH-, as well as SAL-induced PRL release *in vitro*.	Hashizume et al. (2008b)
	1–1 mM	human embryonic kidney (HEK-293), human neuroblastoma (SH-SY5Y) and human glioblastoma (HTZ-146 cells	SAL was the endogenous key substrate of the sodium-independent organic cation transporter (OCT2). OCT2 was preferentially expressed in the dopaminergic regions of the substantia nigra where it co-localized with DAT and TH. SAL exhibited a selective toxicity toward OCT2-expressing cells that was prevented by cyclo(his-pro).	Taubert et al. (2007)
Sigma Aldrich, St. Louis, MO, USA	50–500 μM	human neuroblastoma (SH-SY5Y) cells	SAL treatment caused up-regulation in the levels of c-Jun and phosphorylated c-Jun. The binding activity of NF-κB to DNA was enhanced by SAL in the concentration dependent manner. SAL decreased the levels of the anti-apoptotic protein Bcl-2 and increased pro-apoptotic protein Bax, while enhancing the release of cytochrome-c from mitochondria.	Wanpen et al. (2007)
	0 – 0.8 mM		Exposure to 0.4 mM of SAL resulted in approximately 65% reduction in cell viability. Maximal toxic effect was observed with 0.8 mM of SAL where approximately 80% of cells did not survive.	Copeland et al. (2005)
	0–500 μM	human neuroblastoma (SH-SY5Y) andmice fetal mesencephalic cell	SAL increased the production of reactive oxygen species and significantly decreased glutathione levels and cell viability in SH-SY5Y cells. SAL decreased intracellular ATP levels and induced nuclear condensation in these cells. SAL-induced depletion in cell viability was completely prevented by N-acetylcysteine.	Wanpen et al. (2004)
	100 μM	human neuroblastoma (SH-SY5Y) cell	Both exogenous IGF-1 and IGF-1 gene transfer significantly prevented the SAL-induced cell death and increased cell viability.	Shavali et al. (2003)
	10–200 μM	human melanoma (FRM, MNT and M14) and murine melanoma (B16) cells	SAL enhanced TH activity and melanin production.	De Marco et al. (2002)
	0.01–1000 μM	human embryonic kidney (HEK-293) and mouse neuroblastoma (Neuro-2A) cells	Only 2(N)-methylated isoquinoline derivatives structurally related to MPTP/MPP^+^ are selectively toxic to dopaminergic cells via uptake by the DAT.	Storch et al. (2002)
	1 mM	dopaminergic neuronal (SN4741) cells	SAL induced the moderate ROS activity compared to paraquat, and subsequently activated much lower level of JNK1/2 activity compared to MPP+ and paraquat treatments.	Chun et al. (2001)
	0–500 μM	rat pheochromocytoma (PC12) cells, pBR322 and X174 supercoiled DNA, calf thymus DNA	SAL in combination with Cu(II) induced strand scission in pBR322 and X174 supercoiled DNA, which was inhibited by the copper chelator, reactive oxygen species (ROS) scavengers, reduced glutathione and catalase. Reaction of calf thymus DNA with SAL plus Cu(II) resulted in substantial oxidative DNA damage as determined by 8-hydroxydeoxyguanosine (8-OH-dG) formation. Blockade of the dihydroxyl functional group of SAL abolished its capability to yield 8-OH-dG in the presence of Cu(II).	Jung et al. (2001)
		rat pheochromocytoma (PC12) cells	SAL causes reduced viability, which was exacerbated by Cu^2+^. Although SAL alone could cause apoptotic death in PC12 cells, cells treated with SAL together with Cu^2+^ became necrotic.	Kim et al. (2001)
	0-200 μM	dopaminergic neuronal (RCSN-3) cells	SAL was found to decrease survival in RCSN-3 cells (derived from adult rat substantia nigra) in a concentration-dependent manner (208 μM of SAL induced a 50% survival decrease). *In vitro* oxidation of salsolinol to o-quinone catalyzed by lactoperoxidase gave the quinone methide and 1,2-dihydro-1-methyl-6,7-isoquinolinediol as final products of salsolinol oxidation as determined by nuclear magnetic resonance spectroscopy (NMR) analysis	Martinez-Alvarado et al. (2001)
Synthesized (according to Haber et al. 1993)	1 mM (R- and S-SAL)	mouse anterior pituitary tumor (AtT-20) cells (clone D16v)	SAL bound to the D(2) receptor family, especially to the D(3) receptor with a K(i) of 0.48+/-0.021 μM. S-SAL significantly inhibited the formation of cyclic AMP and the release of beta-endorphin and ACTH in a pituitary cell system.	Melzig et al. (2000)
Sigma Aldrich, St. Louis, MO, USA	0–1000 μM	human neuroblastoma (SH-SY5Y) cells	SAL was cytotoxic to human SH-SY5Y cells via impairment of cellular energy production. The IC_50_ = 34.2 μM (after 72 h) was established for SAL.	Storch et al. (2000)
Synthesized (according to Teitel et al. 1972)	0.1 μM–10 mM (R- and SSAL)		The IC50 values were 540.2 μM for (R)-SAL and 296.6 μM for (S)-SAL.	Takahashi et al. (1997)
Synthetized (according to Haber et al. 1993)	0-500 μM (R- and S-SAL)	mouse anterior pituitary tumor (ArT-20) cells	A significant decrease in the proopiomelanocortin (POMC) gene expression by the S-SAL was noted. The basal secretion of adrenocorticotropin (ACTH) as well as the corticotropin-releasing factor-stimulated ACTH release remained unchanged after R- and S-SAL treatment. It was shown that a reduction of intracellular cAMP level occurred after the treatment of the cells with S-SAL whereas R-SAL did not affect the cAMP production.	Putscher et al. (1995)
Sigma Aldrich, St. Louis, MO, USA	0.001–1 mM	human neuroblastoma (SH-SY5Y) cells	SAL stimulated catecholamine uptake with EC_50_ values of 17 μM and 11 μM, for NA and DA, respectively. At concentrations above 100 μM, SAL inhibited the uptake of NA and DA, with IC_50_ values of 411 μM and 379 μM, respectively.	Willets et al. (1995)
N/A	0.001–10 mM	calf aortic endothelial (BKEz-7) cells	SAL damaged the cultivated calf aortic endothelial cells (cytotoxic effects estimated by cell counting after 72 h treatment with SAL, IC_50_ = 38 μM), especially the mitochondria, and inhibited the respiration measured as inhibition of the oxygen consumption. The damage of endothelial cells was confirmed by the electron microscopy with various disintegrations of mitochondria.	Melzig and Zipper (1993)
**Other** ***in vitro*** **models**				
**Salsolinol source**	**Salsolinol concentration**	**Model**	**Main outcomes**	**References**
Sigma Aldrich, St. Louis, MO, USA	0.1–2 mM	human ceruloplasmin (hCP)	Incubation of hCP with SAL increased the protein aggregation and enzyme inactivation in a dosedependent manner. Reactive oxygen species scavengers and copper chelators inhibited the SALmediated hCP modification and inactivation. The formation of dityrosine was detected in SALmediated hCP aggregates. Amino acid analysis post the exposure of hCP to SAL revealed that aspartate, histidine, lysine, threonine and tyrosine residues were particularly sensitive.	Kim et al. (2016)
	0.01–1 μM	230 μm horizontal slices of CD-1 mice midbrain	SAL was able to excite pVTA DA cells mice treated with α-methyl-p-tyrosine (a DA biosynthesis inhibitor). SAL was needed for ethanol-induced pVTA DA cells activation since neither acetaldehyde nor ethanol was able to excite these neurons in the absence of DA.	Melis et al. (2015)
	0.05–1 mM	horse cytochrome c	Protein aggregation increased in a dose-dependent manner after incubation of cytochrome c with SAL. The formation of carbonyl compounds and the release of iron were obtained in salsolinoltreated cytochrome c. Reactive oxygen species scavengers and iron specific chelator inhibited the SAL-mediated cytochrome c modification and carbonyl compound formation.	Kang (2013)
	0.5 mM	neurofilament-L (NF-L)	NF-L exposure to SAL produced losses of glutamate, lysine and proline residues. Carnosine and anserine were shown to significantly prevent SAL-mediated NF-L aggregation.	Kang (2012)
	0-1000 μM alone or in presence of Cu or Fe	plasmid DNA pBR322 or calf thymus DNA	SAL in the presence of divalent copper induced strand scission and damage in both plasmid and genomic DNA.	Tharakan et al. (2012)
	0.01–1 μM	250–300 μm coronal slices of rat midbrain	SAL excited VTA-dopamine neurons indirectly by activating μ-opioid receptors, which inhibited GABA neurons in the VTA.	Xie et al. (2012)
		200–250 μm coronal slices of rat midbrain	SAL via the activation of presynaptic D1receptors and facilitation of glutaminergic transmission contributed to SAL-induced excitation of pVTA DA neurons.	Xie and Ye (2012)
	5 mM	equine spleen ferritin	The exposure of ferritin to SAL resulted in the generation of protein carbonyl compounds and the formation of dityrosine.	Kang (2010)
	0-0.2 mM	pUC19 plasmid DNA purified from Escherchia coli	SAL/ferritin system-mediated DNA cleavage and mutation was attributed to hydroxyl radical generation via the Fenton-like reaction of free iron ions released from oxidatively damaged ferritin.	Kang (2009)
	0–5 mM	human Cu,Zn-superoxide dismutase	SAL led to inactivation of Cu,Zn-superoxide dismuthase (SOD) in a concentration-dependent manner. Free radical scavengers and catalase inhibited the SAL-mediated Cu,Zn-SOD modification. Exposure of Cu,Zn-SOD to SAL led also to the generation of protein carbonyl compounds.	Kang (2007)
	10, 20, 50 nM	an Fe^3+^–EDTA–H_2_O_2_ complex and a NO–H_2_O_2_ system	The *in vitro* production of the cytotoxic hydroxyl radicals (*OH) was recorded during the autoxidation of SAL.	Nappi et al. (1999)
Synthetized (according to Teitel et al. 1972)	0.05–1 mM (R- and S-SAL; hydrobromide)	pig brain soluble and membrane-bound catechol-O-methyltransferase (COMT)	Kinetic analysis of the O-methylation by S-COMT yielded almost equivalent Km values of 0.138 mM [(R)-SAL] and 0.156 mM [(S)-SAL]. Both enantiomers had similar Vmax values (0.201 and 0.189 nmol min-1 mg protein-1, respectively).	Hötzl and Thomas (1997)
Sigma Aldrich, St. Louis, MO, USA	0–500 μM	øX174 RFI supercoiled DNA, calf thymus DNA, PC12 cells	Incubation of SAL and CuCl_2_ with calf thymus DNA caused strand breaks. SAL in combination with Cu(II) mediated the strand scission in øX174 RFI supercoiled DNA in a time-related manner. SAL induced cell death in cultured PC12 cells, which was exacerbated by Cu(II).	Kim et al. (1997)
Synthesized (King’s College of London, London, UK)	100 μM	male Wistar rat striata synaptosomes	SAL (100 μM) produced the 39.9% inhibition of the [3H]dopamine uptake.	McNaught et al. (1996a)
	0.5–10 mM	intact Wistar rat liver mitochondria	Isoquinoline derivatives may exert mitochondrial toxicity *in vitro* similar to that of MPTP/MPP_+_, however SAL is a weak inhibitor of mitochondrial respiration. Qualitative structure-activity relationship studies revealed that isoquinolinium cations were more active than isoquinolines in inhibiting mitochondrial respiration.	McNaught et al. (1996b)
N/A	0–0.5 mM	microsomal fractions of male Wistar rats livers	Histamine and SAL competitively inhibited the activity of debrisoquine 4-monooxygenase (Ki = 0.31 and 0.43 mM, respectively).	Iwahashi et al. (1993)
Synthetized from salsolidine		human placental MAO A and human liver MAO B	Stereoselective competitive inhibition of MAO (monoamine oxidase) type A was found with the (R)-SAL (Ki = 31 μM), but not MAO type B.	Bembenek et al. (1990)
Synthetized	10-30 μM	liver homogenate (human liver dihydropteridine reductase)	SAL inhibited human liver dihydropteridine reductase non-competitively.	Shen et al. (1982)
Synthesized (according to Craig et al. 1952)	10–200 μg/ml, 333 μg/ml (hydrobromide)	chick biventer cervicis nerve muscle preparation, guinea pig ileum, chick biventer cervicis homogenates	SAL produced agonist effects at muscarinic receptors. In the chick biventer cervicis preparation, SAL (10-200 pg/mL) produced initial twitch augmentation, followed by blockade accompanied by a slowly developing contracture. Responses to exogenous carbachol were unaffected while those to acetylcholine were augmented. The neuromuscular blockade was unable to be reversed by choline, caffeine, physostigmine or tetanus.	Rodger et al. (1979)

Thus, there are also multiple factors that should be considered when trying to compare different studies to determine the ability of salsolinol to cross the blood–brain barrier: the different commercial sources or methods of their laboratory synthesis, the applied doses and enantiomers as well as routes of administration, and the animal species and strains used in the experiments. Some of these aspects are further summarized in Tables 3 and 4. For example, the pharmacokinetics of intravenous (i.v.) and intraperitoneal (i.p.) injections differ from each other—during i.v. injection, the maximum concentration in the blood is reached almost at once and immediately begins to fall; however, after i.p. injection, most of the drug is absorbed by the mesentery veins and then gathered into the portal vein of the liver, similar to oral gavage. While intracerebroventricular drug administration is a method that bypasses the blood–brain barrier and fundamentally differs from the systemic drug administration in terms of pharmacokinetic characteristics to determine the brain tissue concentrations (Cook et al. 2009). However, further research is needed to clarify various aspects of complex salsolinol (among others tetrahydroisoquinoline derivatives) pharmacokinetics.

## Dose-Related Neurobiological Activity of Salsolinol

Maruyama et al. (1995), for the first time, concluded that salsolinol and its derivatives might possess both neurotoxic and neuroprotective activity. Exogenous (R)-salsolinol (40 μM), the isoquinolinium ion (200 μM), and *N*-methyl-(R)-salsolinol (200 μM) reduced in vivo free radical formation and reduced dopamine catabolism. (R)-Salsolinol and the isoquinolinium ion reduced in vitro hydroxyl radical production from dopamine autoxidation. However, on the other hand, *N*-methyl-(R)-salsolinol (40 μM) increased the hydroxyl radical level in the striatum, and the free radical production by its autoxidation was confirmed in vitro. *N*-Methyl-(R)-salsolinol affected neither in vivo dopamine catabolism nor in vitro production of hydroxyl radicals from dopamine (Maruyama et al. 1995). Biphasic effects, either neuroprotective or neurotoxic, of exogenous salsolinol applied as a racemic mixture were confirmed by Możdżeń et al. (2015) in vitro*.* In rat hippocampal cell cultures, the lower investigated dose of salsolinol (50 and 100 μM) was diminished, while its highest dose (500 μM) potentiated the glutamic acid effect on caspase-3 activity. Similar effects were observed for lactate dehydrogenase (LDH) release. In mouse striatum cultures, both the investigated doses of salsolinol (50 and 500 μM) revealed the neuroprotective activity. Authors concluded that salsolinol under physiological conditions could not be neurotoxic (Możdżeń et al. 2015). Our own preliminary, yet unpublished, in vitro results also suggested that the biological action of exogenous salsolinol applied as a racemic mixture, either neurotoxic or neuroprotective, might be indeed dose and time dependent.

Thus, it seems reasonable to wonder whether alcohol consumption should lead to an increased risk of the development of Parkinson’s disease According to Lee et al. (2010), no significant changes in the salsolinol plasma levels or its enantiomeric distribution after acute or chronic ethanol exposure of healthy humans were noted, suggesting that salsolinol may not be a biomarker for ethanol drinking (Lee et al. 2010). Additionally, unfortunately, there is no agreement within the epidemiological studies, and the question regarding whether ethanol consumption (together with smoking and coffee consumption) might be biologically protective or toxic remains a matter of debate. For example, Bettiol et al. recently reported that studies with a prospective design did not support any association between total alcohol intake and PD risk (with two studies finding an increased risk with a moderate alcohol consumption), and the case-control studies were more likely to find protective effects of alcohol on PD risk (Bettiol et al. 2015). Ward et al. highlighted neurochemical pathways related to monoamine oxidases and nitric oxide synthase as those involved in the protective effects of nicotine and ethanol in preventing the development or delay in the progression of Parkinson’s disease (Ward et al. 2008).

Tetrahydroisoquinoline derivatives, which are abundant in nature (Rommelspacher and Susilo 1985), can be divided into catechol and non-catechol structures depending on the biogenic amines participating in a condensation (so-called Pictet–Spengler) reaction. The catechol (1,2 –dihydroxybenzene) moiety occurs widely in nature and is usually associated with anti-oxidant properties. The relatively high anti-oxidant activity of catechol can be explained by the high electron-donating effect of one hydroxyl group to the other (Heijnen et al. 2011, 2002). Salsolinol possesses a catechol moiety; thus, at least theoretically, it might limit excitotoxicity and oxidative stress, which might have clinical implications. However, 1-methyl-1,2,3,4-tetrahydroisoquinoline (a non-catechol derivative) is regarded as one of the most potent neuroprotectants among tetrahydroisoquinolines in the brain. Its neuroprotective effect did not induce the development of tolerance after chronic administration and might restore the function of dopamine neurons, suggesting its clinical relevance (Wąsik et al. 2016; Vetulani and Antkiewicz-Michaluk 2012).

Tetrahydroisoquinoline derivatives, which can mostly be either synthesized endogenously and/or delivered exogenously, can predominantly alter central dopaminergic pathways. Thus, their physiological/pathophysiological role should not be ignored. Multiple factors, such as the dose, stereoisomerism, local bioavailability, local biogenic amine metabolism or even genetic heritage, may determine their biological, possibly opposing, activity.

## 6. Conclusions

The neuromodulatory role of salsolinol is indeed still poorly understood. In general, the available experimental data include many single-center findings for which replication by other researchers has not been reported and might be partly considered plausible. The most scrupulous attention should be paid to the role of the salsolinol’s stereoisomers. Whether salsolinol present in the brain comes solely from local production or, by contrast, there is a significant contribution from the non-cerebral sites or from dietary sources should be established at the earliest convenience.

Salsolinol co-localizes with dopamine-rich regions in the brain. The same should apply for the peripheral tissues, therefore boosting further advances in this field. Because the enteric nervous system and gut microbiota are the major sources of serotonin and dopamine in the human body (Martinucci et al. 2015), the results might be of clinical relevance not only in terms of prodromal Parkinson’s disease but also gastrointestinal and gut-brain axis disorders.
